# Elevated Urinary Levels of Cystatin C and Neutrophil Gelatinase-Associated Lipocalin in Henoch-Schönlein Purpura Patients with Renal Involvement

**DOI:** 10.1371/journal.pone.0101026

**Published:** 2014-06-25

**Authors:** Tao Chen, Yong-hong Lu, Wen-ju Wang, Cai-yun Bian, Xiao-yun Cheng, Yu Su, Pei-mei Zhou

**Affiliations:** 1 Department of Dermatovenereology, Chengdu second people’s hospital, Chengdu, China; 2 Department of Nephrology, Chengdu second people’s hospital, Chengdu, China; 3 Department of Rheumatology, Chengdu second people’s hospital, Chengdu, China; University of São Paulo School of Medicine, Brazil

## Abstract

Henoch-Schönlein purpura (HSP) is a commonest systemic vasculitis in childhood. The long-term prognosis of HSP is determined by the degree of renal involvement. The aim of this study is to search novel clinically applicable biomarkers to evaluate renal involvement in HSP patients. 20 bio-indexes in urine samples were simultaneously screened by antibody array assay. We indicated that urinary levels of cystatin C (Cys C) and neutrophil gelatinase-associated lipocalin (NGAL) in HSP patients with renal involvement were significantly higher than those without renal involvement and healthy controls. Furthermore, ELISA was used to analyze urinary Cys C and NGAL levels in HSP patients with or without renal involvement, atopic dermatitis (AD) patients and healthy controls. Our results demonstrated that urinary Cys C and NGAL levels in HSP patients with renal involvement were significantly elevated, when compared with those without renal involvement, AD patients and control subjects. In addition, by receiver operating characteristic (ROC) curve analysis, we demonstrated that the area under the ROC curve of NGAL (0.789) was larger than that of Cys C (0.692). Taken together, we show firstly that urinary Cys C and NGAL levels is abnormally elevated in HSP patients with renal involvement. We suggest that urinary Cys C and NGAL are novel useful biomarkers of renal involvement in HSP patients.

## Introduction

Henoch-Schönlein purpura (HSP) is a commonest systemic vasculitis in childhood with purpuric rash, arthritis, renal involvement and abdominal pain. It has also been known as an immune complex-mediated disease characterized by circulating immune complexes containing immunoglobulin A (IgA) predominantly depositing on small vessel wall.

The most frequent complication of HSP is renal involvement, which can appear at onset or during the course of this disease. Although most of HSP patients with renal involvement have a good prognosis, some patients do develop end-stage renal disease (ESRD) [1]. Moreover, it has been noticed that HSP is the most prevalent etiology of secondary glomerulonephritis in children [2]. Blood pressure measurement and urinalysis are mandatory when evaluating a child with HSP. However, even if urinalysis is normal at presentation, urinalysis is still needed up to six months after diagnosis as the great majority (97%) of children will have abnormal urine findings within this period [3]. Serum creatinine measurement is also often used to evaluate HSP patients. Unfortunately, serum creatinine is a delayed and insensitive index for the detection of renal involvement in HSP patients.

Thus, it would be interesting to search novel clinically applicable biomarkers to evaluate renal involvement in HSP patients. In this study, urine samples from HSP patients with or without renal involvement were simultaneously screened by using an commercially available antibody array consisting of 20 bio-indexes, which have been identified as potential biomarkers of acute kidney injury. We found that urinary levels of cystatin C (Cys C) and neutrophil gelatinase-associated lipocalin (NGAL) in HSP patients with renal involvement were significantly higher than those without renal involvement and healthy controls. Then the levels of urinary Cys C and NGAL in HSP patients and healthy controls were quantitatively analyzed by enzyme-linked immunosorbent assay (ELISA). Patients with atopic dermatitis (AD), an inflammatory skin diseases without vasculitis, were also enrolled into this study as disease control.

## Materials and Methods

### Patients and control cohorts

Seventy-two patients with HSP who met the diagnostic criteria for HSP [4], 29 patients with AD, together with 51 healthy controls, were enrolled into the study. This study was approved by the Institutional Ethics Committee of Chengdu second people’s hospital. Written informed consent was obtained from all patients or legal guardians on behalf of the children. A detailed history and a complete physical examination were obtained from all patients (patient and control demographics together with detailed clinical information are provided in Tables. 1 and 2). All patients had a minimum follow-up of 6 months from purpuric rash onset. We divided the patients with HSP into two categories, based on with or without renal involvement (HSP1, HSP patients without renal involvement, n = 41; HSP2, HSP patients with renal involvement, n = 31). Renal involvement was defined by the presence of hematuria and/or proteinuria. No statistical differences in age and gender were found among the four groups. 5 ml of first-morning urinary samples were obtained from these HSP patients after purpura onset and then stored at −80°C until use.

**Table 1:** Cohort demographics.

	HSP1	HSP2	AD	Controls
Number	41	31	29	51
Age (Range)	13 (5–28)	14 (5–25)	14 (6–22)	14
Sex	21 M/20F	16 M/15F	14 M/15F	25 M/26F

**Table 2:** Clinical characteristics of patients with HSP.

Symptoms and signs	n.	%
Cutaneous palpable purpuric rashPrevious URIInternal organ involvementArthritis and/or arthralgiaRenal involvementAbdominal pain	722358333117	10031.980.651.543.123.6

URI, upper respiratory tract infection.

### Urinary biomarkers antibody array assay

Bio-indexes present in the urinary samples from part of HSP1 group, HSP2 group and control group (all n = 6) were analyzed by using the Human Acute Kidney Injury Antibody Array (RayBiotech, Inc., Norcross, GA, USA) following the protocols of the manufacturers.

### Measurement of urinary levels of Cys C and NGAL

Urinary levels of Cys C and NGAL (Boster Biosciences Co., Wuhan, China) in different groups were quantitated with commercially available ELISA kits according to the manufacturer’s instructions.

### Statistical analysis

Each experiment was performed at least three times. All results are expressed as mean ± SD, Statistical differences between groups were determined according to one-way analysis of variance (ANOVA), Kruskal-Wallis test and Mann-Whitney U Test. Correlation coefficients were obtained by Spearman tests. Receiver operating characteristic (ROC) curve was generated and the area under the curve was calculated to analyze and describe the accuracy of Cys C and NGAL as biomarker. P<0.05 was considered statistically significant.

## Results

### Increased urinary levels of Cys C and NGAL in HSP patients with renal involvement

**Figure 1:**
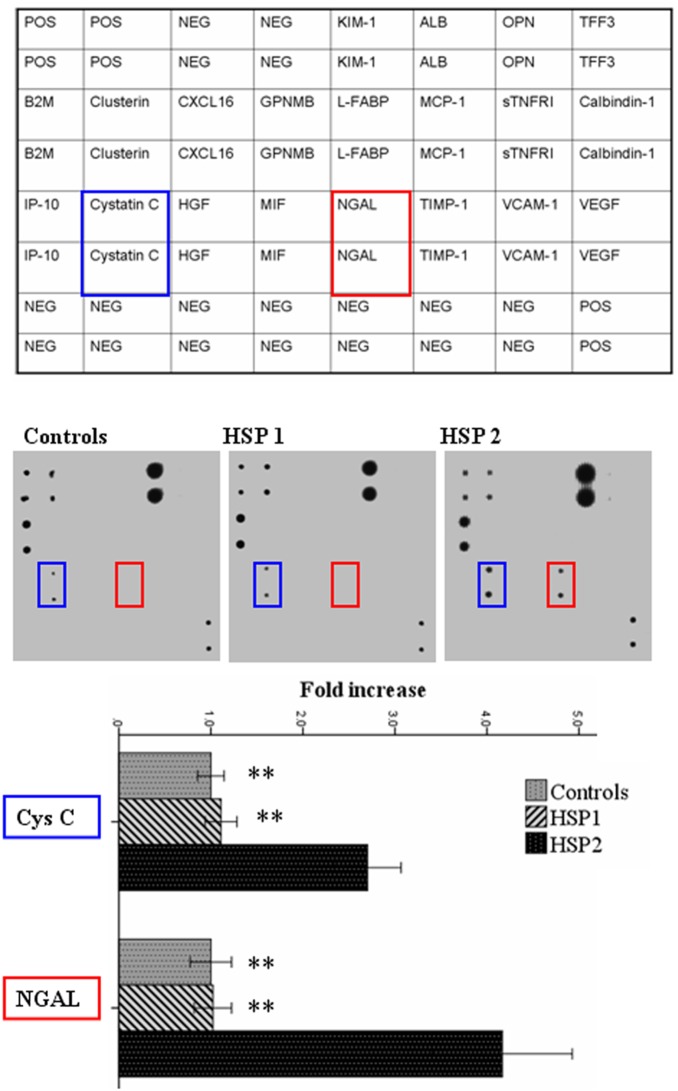
Urinary bio-indexes levels in HSP patients. 20 bio-indexes were simultaneously measured in the urinary samples from HSP patients with or without renal involvement and healthy controls by antibody array assay. Representative membrane antibody array and relative elevated bio-indexes levels in the urinary samples from HSP patients with renal involvement were shown. Values are expressed as Mean ± SD; one-way analysis of variance n = 6. **P<0.01, compared with HSP2 group. HSP1, HSP without renal involvement; HSP2, HSP with renal involvement. POS, Positive Control; NEG, Negative Control; KIM-1, Kidney Injury Molecule-1; ALB, Albumin; OPN, Osteopontin; TFF3, Trefoil factor 3; B2M, β2-Microglobulin; GPNMB, Glycoprotein non-metastatic melanoma protein B; L-FABP, Liver Fatty-Acid Binding Protein; MCP-1, Monocyte chemoattractant protein 1; sTNFRI, Soluble tumor necrosis factor receptor I; IP-10, IFN-γ-inducible protein 10; HGF, Hepatocyte Growth Factor; MIF, Macrophage migration Inhibitory Factor; TIMP-1, tissue inhibitor of metalloproteinases-1; VCAM-1, vascular cell adhesion molecule–1; VEGF, vascular endothelial growth factor.

First, 20 bio-indexes were simultaneously detected by using Human Acute Kidney Injury Antibody Array. Fig. 1 provides an overview of these bio-indexes levels in the urinary samples from part of HSP1 group, HSP2 group and control group. We demonstrated that the urinary levels of albumin, β2-microglobulin, Cys C and NGAL in the urinary samples from HSP2 group were detectable. Among them, only Cys C and NGAL urinary levels in HSP2 group were significantly higher than those in HSP1 group (both p<0.001) and control subject (both p<0.001). Urinary albumin and β2-microglobulin levels were elevated (not statistically significant) in HSP2 group, when compared with those in HSP1 group (both p>0.05) and control subject (both p>0.05 and data not shown). In addition, there were no significant changes in urinary levels of these bio-indexes between HSP1 group and control group (Fig. 1 and data not shown).

**Figure 2:**
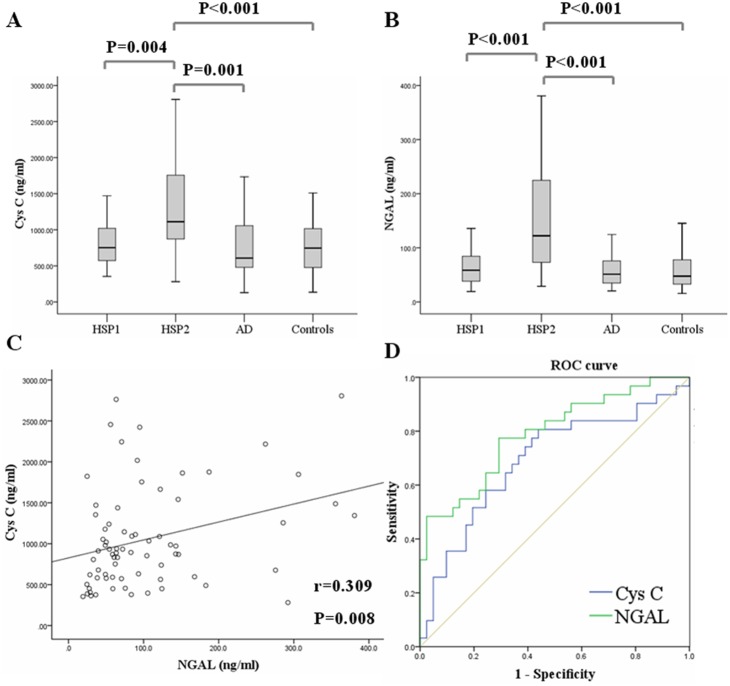
Elevated urinary levels of Cys C and NGAL in HSP patients with renal involvement. Urinary samples were obtained from HSP patients with (HSP1 group, n = 41) or without (HSP2 group, n = 31) renal involvement, AD patients (n = 29) and control subjects (n = 51). Urinary levels of Cys C and NGAL were determined by ELISA. Urinary Cys C (A) and NGAL (B) levels in HSP2 group were significantly elevated, when compared with those in HSP1 group, AD group and control group. P values are based on the Mann-Whitney U Test. (C) A positive correlations were found between urinary Cys C and NGAL levels in HSP patients by Spearman tests. (D) ROC curves for Cys C and NGAL are shown. The area under the ROC curve of NGAL was larger than that of Cys C (0.789 VS 0.692).

In order to further establish the results from antibody array assay, ELISA was used to analyze Cys C and NGAL levels in the urinary samples from HSP1 group, HSP2 group, AD group and control group. As shown in Fig. 2A and 2B, urinary levels of Cys C and NGAL in HSP2 group (1270.58±661.94 ng/ml; 154.13±106.61 ng/ml, respectively) were significantly elevated, when compared with those in HSP1 group (891.81±523.14 ng/ml, p = 0.006; 66.03±35.99 ng/ml, p<0.001, respectively), AD group (809.52±518.91 ng/ml, p = 0.004; 65.75±46.32 ng/ml, p<0.001, respectively) and control group (803.87±566.4 ng/ml, p = 0.006; 64.62±45.87 ng/ml, p<0.001, respectively).

We also investigated the relationship of Cys C and NGAL urinary levels with different clinical manifestation in HSP patients (HSP1 and HSP2). However, there were no significant difference in Cys C and NGAL urinary levels between HSP patients with or without arthritis, abdominal pain, and upper respiratory tract infection (data not shown).

### Association of urinary levels of Cys C and NGAL in HSP patients

Furthermore, correlation analysis was used to investigate the relationships of Cys C and NGAL. As presented in Fig. 2C, there was a significant positive correlation (r = 0.309, p = 0.008) between Cys C and NGAL levels in the urinary samples from HSP patients (HSP1 and HSP2).

### Urinary Cys C and NGAL for diagnosis of renal involvement in HSP patients

The ROC curve was used to evaluate the sensitivity and specificity to discriminate renal involvement for various cut-off values of urinary Cys C and NGAL. ROC curves for Cys C and NGAL are shown in Fig. 2D, the area under the ROC curve of NGAL (0.789) was larger than that of Cys C (0.692). The cut-off levels of Cys C and NGAL with optimal sensitivity and specificity were 1102.36 ng/ml (sensitivity 51.6%; specificity 80.5%) and 71.45 ng/ml (sensitivity 77.4%; specificity 70.7%) respectively.

## Discussion

HSP is an IgA related immune complex-mediated disease in childhood and is also known as a classic leucocytoclastic vasculitis characterized by perivascular leukocyte infiltrates. The long-term prognosis of HSP is determined by the degree of renal involvement, which can manifest with haematuria, with or without proteinuria, and occasionally with nephritic or nephrotic syndrome, or with renal failure.

Over the years, a great deal of research has been preformed to investigate urinary biomarkers for the diagnosis or prediction of renal injury [5], [6]. However, only a few studies have been conducted on HSP. Cys C is a 13-kDa endogenous cysteine proteinase inhibitor. It is freely filtered by the glomerulus, reabsorbed and catabolized by the tubules, and identified as a promising marker of renal dysfunction [7] ^.^ NGAL is a 25-kDa protein produced by neutrophils and various epithelial cells, including renal tubular cells. It is up-regulated in the urine after ischemic renal injury and has been identified as a powerful early marker of acute kidney injury [8], [9]. In this study, by using membrane antibody array including 20 bio-indexes, we indicated that urinary levels of Cys C and NGAL were abnormally elevated in HSP patients with renal involvement. Furthermore, the changes of Cys C and NGAL in HSP patients with renal involvement were established by ELISA. In addition, by ROC curve analysis, we demonstrated that both Cys C and NGAL are useful for diagnosis of renal involvement in HSP patients. We also noticed that NGAL can be up-regulated in acute bacterial infections [10]. However, in this study, no significant change in urinary NGAL levels was determined between HSP patients with or without upper respiratory tract infection (data not shown).

Taken together, this study provides first observations on the changes of urinary Cys C and NGAL levels in patients HSP. We suggest that urinary Cys C and NGAL are promising as novel non-invasive biomarkers useful for early diagnosis of renal involvement in HSP patients. A limitation of our study was the relatively small sample size. Although our results have significant implications, further research with large sample size is still needed. Also, it is necessary to investigate whether the urinary levels of Cys C and NGAL are restored to normal levels in convalescent stage of HSP.
